# The effect of seasonal and extreme floods on hospitalizations for Legionnaires’ disease in the United States, 2000–2011

**DOI:** 10.1186/s12879-022-07489-x

**Published:** 2022-06-15

**Authors:** Victoria D. Lynch, Jeffrey Shaman

**Affiliations:** Department of Environmental Health Sciences, Columbia Mailman School of Public Health, Columbia University, 722 W. 168th St, New York, NY 10032 USA

**Keywords:** Legionnaires’ disease, Flooding, Extreme storms

## Abstract

**Background:**

An increasing severity of extreme storms and more intense seasonal flooding are projected consequences of climate change in the United States. In addition to the immediate destruction caused by storm surges and catastrophic flooding, these events may also increase the risk of infectious disease transmission. We aimed to determine the association between extreme and seasonal floods and hospitalizations for Legionnaires’ disease in 25 US states during 2000–2011.

**Methods:**

We used a nonparametric bootstrap approach to examine the association between Legionnaires’ disease hospitalizations and extreme floods, defined by multiple hydrometeorological variables. We also assessed the effect of extreme flooding associated with named cyclonic storms on hospitalizations in a generalized linear mixed model (GLMM) framework. To quantify the effect of seasonal floods, we used multi-model inference to identify the most highly weighted flood-indicator variables and evaluated their effects on hospitalizations in a GLMM.

**Results:**

We found a 32% increase in monthly hospitalizations at sites that experienced cyclonic storms, compared to sites in months without storms. Hospitalizations in months with extreme precipitation were in the 89^th^ percentile of the bootstrapped distribution of monthly hospitalizations. Soil moisture and precipitation were the most highly weighted variables identified by multi-model inference and were included in the final model. A 1-standard deviation (SD) increase in average monthly soil moisture was associated with a 49% increase in hospitalizations; in the same model, a 1-SD increase in precipitation was associated with a 26% increase in hospitalizations.

**Conclusions:**

This analysis is the first to examine the effects of flooding on hospitalizations for Legionnaires’ disease in the United States using a range of flood-indicator variables and flood definitions. We found evidence that extreme and seasonal flooding is associated with increased hospitalizations; further research is required to mechanistically establish whether floodwaters contaminated with *Legionella* bacteria drive transmission.

**Supplementary Information:**

The online version contains supplementary material available at 10.1186/s12879-022-07489-x.

## Background

Legionnaires’ disease is among the most severe and costly waterborne illnesses in the United States, where it is responsible for an estimated 15% of all deaths related to waterborne infectious disease [1] and between 3 and 9% of all cases of community-acquired pneumonia [2, 3]. Legionnaires’ disease was so named in 1977, when a cooling tower contaminated with the bacteria was found to be the cause of a pneumonia outbreak among guests at a hotel [4], and its incidence has substantially increased since 2000 [5, 6]. Outbreaks of Legionnaires’ disease have decreased over the last 40 years, however, with only 4% of reported cases since 2000 linked to a cluster [7]. Over 80% of cases are sporadic [8], and the source of infection is never identified for the majority of these cases. Legionnaires’ disease cases typically peak in late summer or early fall, and this consistent seasonality suggests that environmental factors affect transmission [9, 10] and may help explain the origin of these sporadic infections.

Environmental conditions affect the proliferation of *Legionella* bacteria in lakes, streams, and estuaries [11, 12], and the contamination events that may lead to disease transmission [13, 14]. The bacteria are abundant in aqueous environments [15] and survive by parasitizing amoebae, including many that persist in environmental biofilms [16, 17]. The bacteria optimally grow in wet, warm conditions (between 25 and 42 °C) and flourish in sessile biofilm communities [18, 19] in the natural and built environment [20, 21]. Environmental events that mobilize biofilms may be an important driver of infection by increasing the bacterial load in plumbed water [22–24], water used for industrial processes [25, 26], and surface water where direct exposure can occur [27]. Susceptible individuals can become infected by inhaling aerosolized bacteria from these contaminated water sources.

Previous studies have found positive associations between cases of Legionnaires’ disease and rainfall [28–31], relative humidity [10, 32], and streamflow [33], and inconsistent associations with proximity to rivers or river height [11, 33]. Temperature has been positively associated with cases in several studies, though its effect is often attenuated when adjusting for other seasonal factors [10, 30]. While many of these hydrometeorological variables are associated with flood events, the relationship between Legionnaires’ disease and flooding has not been formally evaluated. Flooding is known to mobilize bacteria-rich biofilms in water bodies [34, 35], which may lead to increased bacterial colonization of the built environment. Churning flood waters may also lead to the direct aerosolization of bacteria and increased risk of exposure for individuals close to flood waters.

Flooding during extreme storms may be of particular concern because high winds and storm surges can damage or overwhelm the water treatment infrastructure necessary to address contamination events [36, 37]. The effect of extreme floods on waterborne infectious diseases has not been systematically examined in the US; rather, it has only been assessed after specific storm events (e.g. Superstorm Sandy [38, 39], Hurricane Katrina [40]). Increased incidence of intestinal illness has been reported after major storms, however, and post-storm microbiological analyses have found high concentrations of pathogenic bacteria in floodwater [37, 41].

Floods can be measured with a range of hydrometeorological variables and those that best describe extreme or seasonal events often vary by region to reflect local hydroclimatology, geography, and the built environment [42, 43]. These factors determine the conditions under which a flood occurs and help explain, for example, how a single heavy precipitation event can lead to a devastating flash flood in an urban area with a small watershed, whereas the same amount of precipitation has no effect in a rural area with a large drainage basin [44]. Precipitation has traditionally been the primary variable used to determine flood magnitude; however, recent research has demonstrated that soil moisture, snowmelt, and precipitation excess might better characterize flooding in many regions [45]. Most studies that have examined the association between floods and health outcomes have used a single hydrologic indicator [46, 47] or observed storms records [48]. Given that floods cannot be defined by the same set of hydrometeorological variables across all locations, this approach does not allow for the identification of all major flood types in the US (i.e. river, coastal, and flash floods as well as flooding after cyclonic storms).

Understanding the association between Legionnaires’ disease infections and flood events is particularly important given that the severity of flooding is predicted to increase in conjunction with rising temperatures [49, 50]. The severity and timing of river floods is projected to increase due to earlier snowmelt and more intense precipitation [51, 52]. The number of major, billion-dollar floods has increased by 5% each year in the US since 1980 [53]; this is a trend that is likely to continue under future global warming, as more severe cyclonic storms and coastal flood events are projected to occur in the coming decades [54].

In this study, we used nonparametric and generalized linear mixed models to determine the effect of extreme and seasonal floods on hospitalizations for legionnaires’ disease across the US. Previous research has examined the association between single hydrometeorological variables and cases, but a thorough examination of the effect of flooding on legionnaires’ disease has not been conducted. Earlier studies have also been limited to small geographic regions, primarily in the northeastern US, whereas this study includes hospitalizations from 25 states throughout the US. Using this national dataset, we have quantified the effects of extreme and seasonal floods, measured using multiple flood-indicator variables, on hospitalizations for Legionnaires’ disease across the US.

## Methods

### Data

#### Hospitalization data

Legionnaires’ disease infections occur primarily among older or immunocompromised individuals, and an estimated 97% of identified cases are hospitalized [1, 18]. We used the National Inpatient Sample (NIS) from the Healthcare Cost and Utilization Project (HCUP) to identify Legionnaires’ disease hospitalizations between 2000 and 2011 throughout the US. The NIS is the largest publicly available all-payer inpatient database in the US; it captures 20% of hospitalizations per year and is designed to be representative of all hospitalizations nationwide. We identified infections by ICD-9 code (482.84) and found the monthly Legionnaires’ disease hospitalization count for each hospital. We restricted our analysis to hospitals that contributed at least 4 years of data to the NIS dataset, provided monthly counts of hospitalizations, and reported their geographic location.

Hospitals that reported no Legionnaires’ disease cases were excluded from the analysis because the absence of cases could indicate that *Legionella* were not present in environmental or household water sources in that region, or because a hospital was not testing for Legionnaires’ disease among patients with pneumonia. Many hospitals reported only one case during the study period; as a sensitivity analysis, we repeated the analyses using several case count thresholds to further restrict the included hospitals. We created subsets of our hospitalization data containing hospitals with at least 1, 5, 10, 15, and 20 Legionnaires’ disease cases during the study period; all of the analyses were repeated with these case count threshold datasets.

The NIS includes the location of the reporting hospital, but not the cases’ residential locations. To address the possibility of misclassification bias, given that the flood data are associated with the location of the hospital, we matched the hospitals to Hospital Service Areas (HSA) provided by the Dartmouth Atlas of Healthcare [55]. The HSA is the catchment area for each hospital and includes the zip codes where most Medicare patients receive care from the given hospital. We repeated the analyses using flood data associated with catchment area, instead of the hospital location, as a sensitivity analysis to assess the consistency of our findings.

#### Flood data

Flooding can be characterized by several hydrometeorological variables, and we used multiple flood-indicator variables to account for the range of flood-types found across the study sites (e.g. river floods, coastal floods, flash floods), and to distinguish between extreme and seasonal events. Precipitation, soil moisture, and surface runoff data were obtained from the NASA/ NOAA North American Land Data Assimilation System 2 (NLDAS-2) forcing dataset and were aggregated from an hourly temporal resolution to mean monthly values for each hospital location [56]. We used the United States Geological Survey (USGS) National Water Information System to find the stream gages closest to each hospital, for those that had a stream gage in the same zip code, and obtained daily median and maximum stream discharge measurements, which were aggregated to monthly means [57].

Data on flooding associated with tropical cyclones were obtained from the NOAA Storm Event Database, which tracks the location, type, and severity of named storms in the Atlantic Storm Basin [58, 59]. For each named storm that occurred during the study period, we extracted county-level data on: 1) storm-related precipitation, 2) reported flooding, and 3) distance from the storm track. Exposure to each of these extreme flood-related indicators was assessed for each hospital and month in the study period.

### Statistical analysis

#### Extreme floods associated with cyclonic storms

Two methods for identifying extreme floods were used to account for the range of flood types that occur in the US. In the first approach, we defined extreme floods as those associated with named cyclonic storms, and restricted the dataset to the hospitals that experienced these storms and to the months of the Atlantic Basin storm season (June – November).

We modeled the association between Legionnaires’ disease hospitalizations and extreme storm-related floods using a negative binomial generalized linear mixed model (GLMM) framework to account for the over-dispersed hospitalization data. The counties with HCUP-contributing hospitals were categorized as exposed or unexposed to storms for each month during the storm season between 2000 and 2011. A county was considered exposed if it was within 150 km of the storm track and unexposed if it was outside of that range. In addition to the binary exposure variable, we assessed storm-related precipitation and proximity to the storm track as continuous variables and as categorical variables grouped by quartile.

The model included a binary location variable to assess differences between rural and urban hospitals and hospital-specific monthly discharges as an offset to obtain the rate of Legionnaires’ disease hospitalizations. We also included hospital-specific random intercepts nested within state-specific random intercepts to account for underlying differences in hospitalization policies (e.g. testing, reporting, and admitting practices) as well as state-level responses to extreme events (Additional file 1: Model S1). The storm-related variables were modeled separately and jointly, and model fit was assessed using the Akaike Information Criterion (AIC). To assess the consistency of our findings, this analysis was repeated for each Legionnaires’ disease case threshold to determine whether a storm in the preceding month was associated with hospitalizations.

#### Extreme floods associated with anomalous hydrometeorology

In the second analysis, we classified months with anomalously high precipitation, soil moisture, surface runoff, or streamflow discharge as those with extreme flooding. For each hospital, we found the months with mean hydrometeorological variables above the 95^th^ percentile and averaged the number of Legionnaires’ disease hospitalizations in this “extreme group”. We compared the hospitalizations in the extreme group to a bootstrapped distribution of monthly Legionnaires’ disease hospitalizations.

The bootstrap generated a sampling distribution by randomly selecting 5% of months in the time series, with replacement, averaging the number of hospitalizations in those months, and then repeating the process 10,000 times. To control for seasonality, the sample was selected from the same range of months as those included in the extreme group for each hospital (i.e. if the extreme group for a given hospital did not include hospitalizations for November, then other November months in that hospital’s time series were not selected during the bootstrapping process). The probability of the Legionnaires’ disease hospitalizations in the extreme group was determined by comparison to the empirical cumulative distribution generated by the bootstrap. The bootstrap process was repeated for the all of the hydrometeorological flood indicators and for each case threshold.

#### Seasonal floods

In a third analysis, we used a multimodel inference approach to determine the effect of seasonal flood indicators on Legionnaires’ disease hospitalizations for the whole time series, not restricted to months with extreme floods or during the Atlantic Basin hurricane season. Multimodel inference was conducted on candidate models that varied only in the explanatory hydrometeorological variables, but that otherwise had the same structure. All combinations of standardized precipitation, soil moisture, surface runoff, and observed flood count were included in the candidate models; temperature was also included, given that the growth of *Legionella* has been associated with temperature seasonality. The models also included terms to control for seasonal and secular trends and a random intercept for each hospital (Additional file 1: Model S2). The streamflow variables were excluded from this analysis due to missing data for hospitals that were not near USGS stream gages.

We used the log likelihood and number of parameters to calculate the Akaike weight for each model. The models were ranked by weight, and the top models, the smallest number of models whose weights added to 0.90, were selected as the best-fitting models. Among the top models, variable weight importance for the hydrometeorological and temperature variables was determined. Cross-validation was performed by removing 20% of the data and conducting multimodel inference on the remainder; this process was iterated 1,000 times to evaluate the consistency of the weights and effect estimates, and to compare them to the top full models. These analyses were repeated for each Legionnaires’ disease case threshold.

## Results

**Fig. 1:**
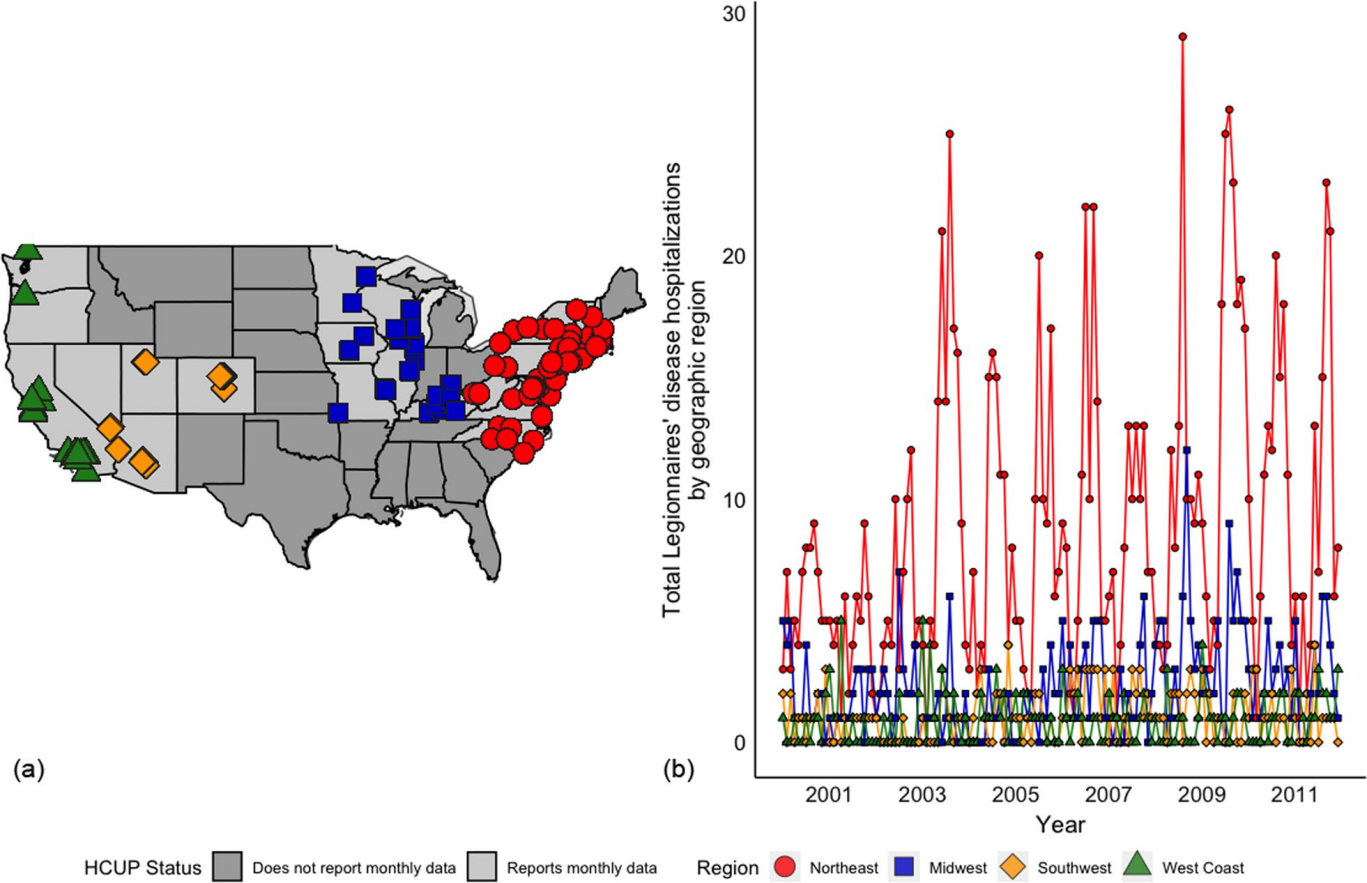
**a** The 75 hospitals in the HCUP dataset with a minimum of 10 total Legionnaires’ disease cases; dark gray states are those that do not participate in HCUP or do not provide monthly data. **b** Total Legionnaires’ disease hospitalizations among the included hospitals between 2000 and 2011 by geographic region

There were 1,376 Legionnaires’ disease hospitalizations between 2000 and 2011 at the 75 hospitals that met our inclusion criteria for the primary analysis (Fig. 1a). Most of these hospitals were large facilities (65.4%) and located in urban areas in the Northeast (66.2%) or Midwest (16.8%). The number, size, and geographic breakdown of the hospitals was relatively consistent across years in the study period, with the exception of 2008 when there were no rural hospitals in the dataset (Table 1). The rural/urban location and hospital bed-size variables were not included in the 2011 HCUP dataset, but the mean annual discharge and geographic region breakdown for this year are consistent with previous years.

**Table 1:** Description of hospitals from the HCUP dataset included in the primary analysis, 2000—2011

Year	2000	2001	2002	2003	2004	2005	2006	2007	2008	2009	2010	2011	Overall.^a^
Number of Hospitals	36	24	36	34	29	30	37	29	38	32	33	31	
Number of LD Cases	92	55	83	133	90	88	139	100	149	182	141	124	1376
Hospital Location (%)													
Rural	11.1	8.3	11.1	5.9	3.4	3.3	8.1	3.4	0	6.2	9.1	-	6.4
Urban	88.9	91.7	88.9	94.1	96.6	96.7	91.9	96.6	100	93.8	90.9	-	93.6
Hospital Bedsize (%)													
Small	8.3	8.3	16.7	11.8	17.2	10	5.4	3.4	5.3	12.5	9.1	-	9.8
Medium	33.3	29.2	22.2	35.3	13.8	20	21.6	24.1	34.2	18.8	18.2	-	24.9
Large	58.4	62.5	61.1	52.9	69	70	73	72.5	60.5	68.7	72.7	-	65.4
Geographic Region (%)													
Northeast	63.9	70.8	61.1	73.5	72.4	66.7	62.2	69	57.9	71.9	63.6	64.5	66.2
Midwest	25	16.7	25	8.8	6.9	13.3	16.2	13.8	21.1	18.8	15.2	19.4	16.8
Southwest	8.3	8.3	8.3	11.8	17.2	13.3	16.2	13.8	18.4	6.2	12.2	9.7	12.3
West Coast	2.8	4.2	5.6	5.9	3.5	6.7	5.4	3.4	2.6	3.1	9	6.5	4.7
Mean Annual Discharge (SD)	20,600 (12,600)	22,600 (12,800)	19,700 (9,620)	21,400 (14,300)	24,900 (13,900)	23,700 (11,000)	24,000 (15,800)	27,600 (13,500)	25,200 (11,200)	25,500 (14,400)	21,900 (13,200)	24,900 (11,200)	23,300 (13,100)
Number of Hospitals	58.4	62.5	61.1	52.9	69	70	73	72.5	60.5	68.7	72.7	-	65.4

^a^75 hospitals were included in the primary analysis, each of which contributed at least 4 years of data; the number per year refers to the number, out of the 75, that contribute in that given year

Seasonality and secular trends in hospitalizations varied by geographic region (Fig. 1). In the Northeast and Midwest, hospitalizations increased between July and October, peaking in August, and also increased over time (Fig. 1b). In the Southwest, hospitalizations exhibited an attenuated seasonality, with increased hospitalizations between March and October, and fluctuated over time. There was no clear seasonal or secular trend in hospitalizations among the hospitals in western states.

The hospital characteristics varied considerably across the datasets with different case count thresholds used in the secondary analysis. The subset of hospitals with at least one Legionnaires’ disease case included 378 hospitals, with many located on the West Coast (17.6%) and in rural areas (23.1%) (Additional file 1: Table S1). At higher case count thresholds, the included hospitals on average had larger bed capacity and were concentrated in urban areas in the Northeast; among the 15-case and 20-case threshold hospitals, none were from rural areas or located on the West Coast (Additional file 1: Table S1). Seasonal and secular trends were consistent across the different case count thresholds.

**Fig. 2:**
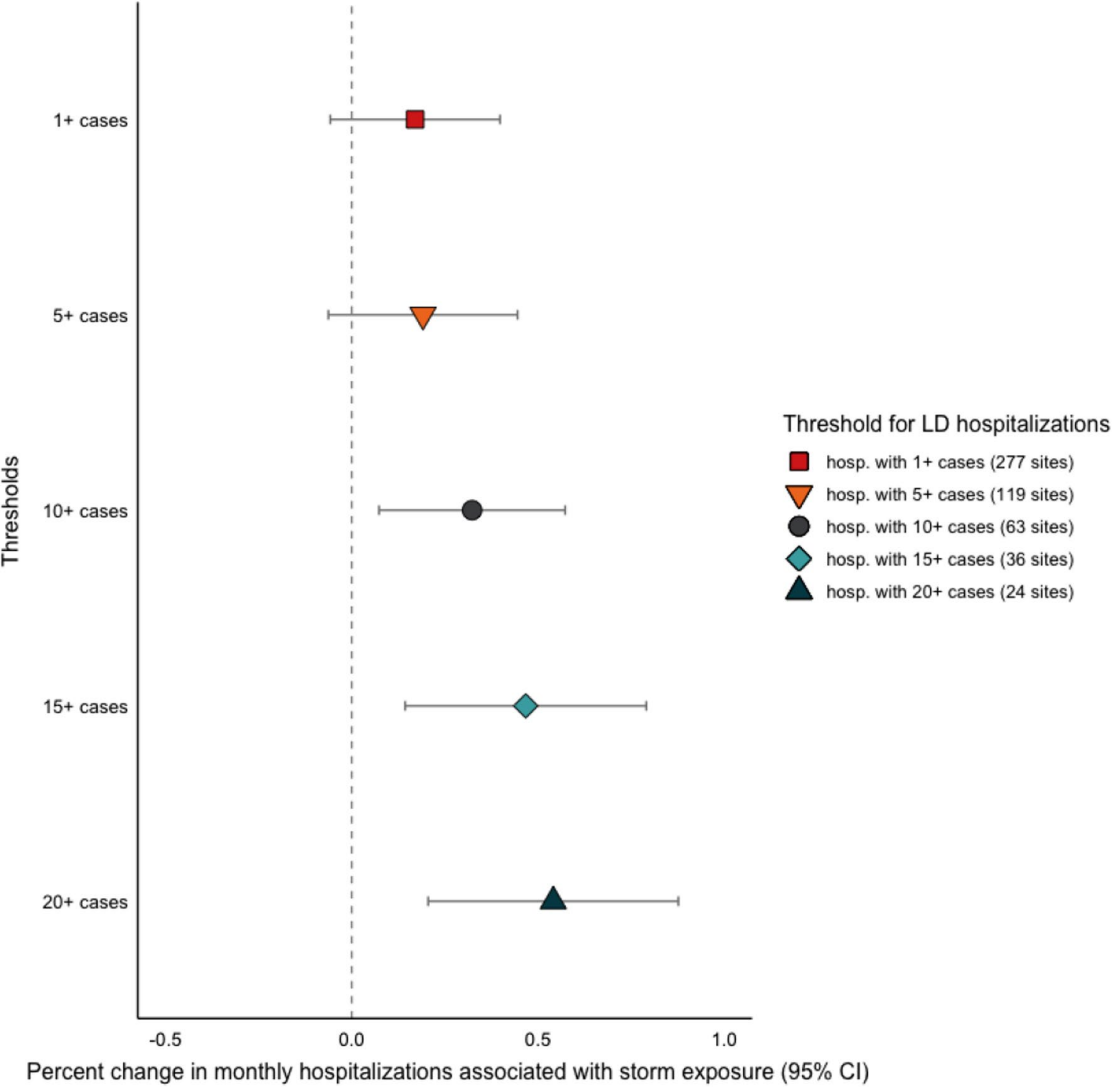
Change in monthly Legionnaires’ disease hospitalizations among hospitals that experienced a cyclonic storm in the same month compared to hospitals that did not experience a storm; analysis was restricted hospitals in regions that experience cyclonic storms from the Atlantic storm basin and to the months of the Atlantic storm season (June–November). Symbols represent the effect estimates from models using the different Legionnaires’ disease case count thresholds

Fifteen named hurricanes or tropical cyclones affected counties with hospitals included in the dataset (Additional file 1: Table S2). Among the hospitals that experienced these storms, there was a significant increase in Legionnaires’ disease hospitalizations during months with a storm compared to months during the Atlantic storm season when a storm did not occur (Fig. 2). There was a 32% increase in monthly Legionnaires’ disease hospitalizations among hospitals that experienced a cyclonic storm compared to those that did not. This association was consistent across the case count thresholds, though it was insignificant in the 1-case and 5-case subsets and stronger in the 15-case and 20-case subsets, where there was a 46% and 54% increase in hospitalizations, respectively, in months with a cyclonic storm compared to those without storms (Fig. 2).

The intensity of storm-related precipitation and proximity to the storm track were not significantly associated with Legionnaires’ disease hospitalizations (Additional file 1: Fig. S1). Hospitals that experienced the most intense storm-related precipitation (quartile 4 of maximum rainfall) had an increase in hospitalizations compared to hospitals that did not experience storm-related precipitation, but the difference was insignificant (Additional file 1: Fig. S1a). Among the hospitals in the 15-case and 20-case subsets, however, this association was significant; hospitalizations increased by 81% and 90%, respectively, with moderate storm-related precipitation (quartile 3 of maximum rainfall) (Additional file 1: Fig. S1a). Proximity to the storm track was not associated with hospitalizations for any of the case count thresholds (Additional file 1: Figure S1b). The sensitivity analysis with storm data aggregated to each hospital’s catchment area yielded results consistent with the primary analysis. There was a 50% increase in monthly Legionnaires’ disease hospitalizations among hospitals in HSAs that experienced a cyclonic storm compared to those that did not (Additional file 1: Fig. S2a), and no significant association with precipitation intensity (Additional file 1: Fig. S2b) or proximity to the storm track (Additional file 1: Fig. S2c).

**Fig. 3:**
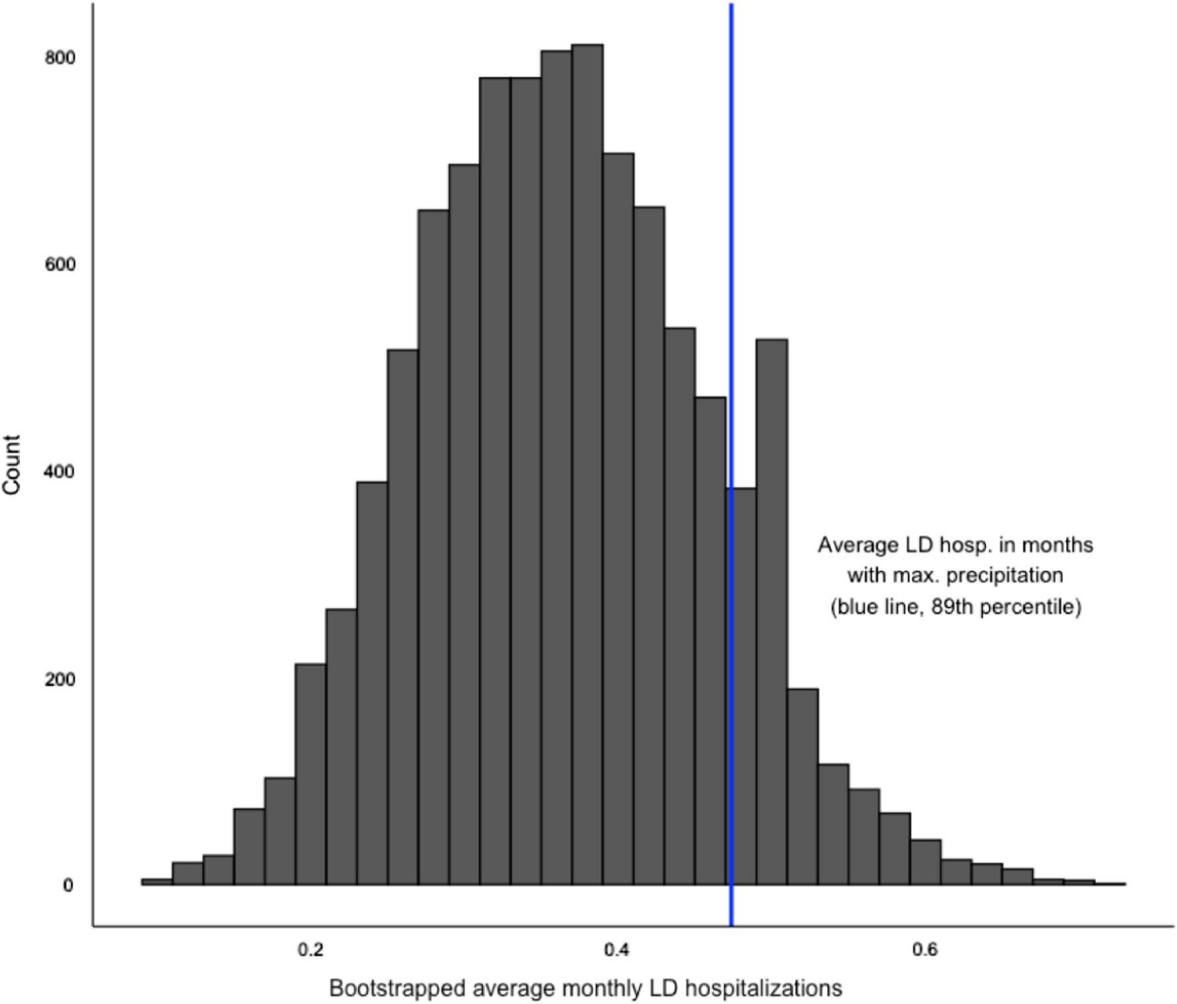
Among the 75 hospitals with at least 10 cases, the average number of Legionnaires’ disease hospitalizations in months with extreme precipitation is in the 89th percentile of the bootstrapped distribution of monthly Legionnaires’ disease hospitalizations

The average number of Legionnaires’ disease hospitalizations in months with extreme precipitation was in the 89^th^ percentile of the bootstrapped distribution (Fig. 3), which was substantially higher than the average number of hospitalizations for all other causes in the same months. The strength of this association increased among the 15-case and 20-case threshold subsets to the 92nd and 94th percentiles, respectively (Table 2). Across all case-count thresholds, Legionnaires’ disease hospitalizations in months with extreme runoff, soil moisture, or temperature did not significantly vary from the bootstrapped averages (Table 2). These findings are supported by the sensitivity analysis using meteorological data aggregated to the hospitals’ catchment areas; the average number of hospitalizations in months with extreme precipitation was in the 84th percentile and increased to the 91st and 93rd percentiles among the higher case thresholds (Additional file 1: Table S3).

**Table 2:** Percentile of average monthly hospitalizations in months with extreme meteorological conditions compared to bootstrapped distribution of average monthly hospitalizations

Hospitalization Threshold	Precipitation	Runoff	Soil Moisture	Temperature
1 + Case	0.87	0.57	0.38	0.52
5 + Cases	0.80	0.60	0.29	0.51
10 + Cases	0.89	0.58	0.50	0.46
15 + Cases	0.92	0.76	0.60	0.47
20 + Cases	0.94	0.70	0.60	0.56

The hydrometeorological flood-indicator variables exhibited seasonal patterns that varied by geographic region (Additional file 1: Fig. S3). Precipitation typically peaked between June and September in the Northeast, Midwest, and Southwest, whereas along the West Coast it was driest during the summer and peaked in December or January (Additional file 1: Fig. S3a). Soil moisture seasonality was consistent across the US, with maxima occurring in January or February and minima mid-summer, but the range varied by region (Additional file 1: Fig. S3b). In the Northeast and Midwest, monthly soil moisture was relatively stable, whereas in the Southwest and on the West Coast there was a steep decline in soil moisture during the summer. Surface runoff exhibited the most distinct seasonality by region; on the West Coast it peaked during the winter, coinciding with the precipitation peaks, whereas in the Northeast surface runoff peaked in late spring, prior to the precipitation peak. Many areas in the Midwest experienced two peaks, one in the early spring and one in the later summer (Additional file 1: Fig. S3c).

**Fig. 4:**
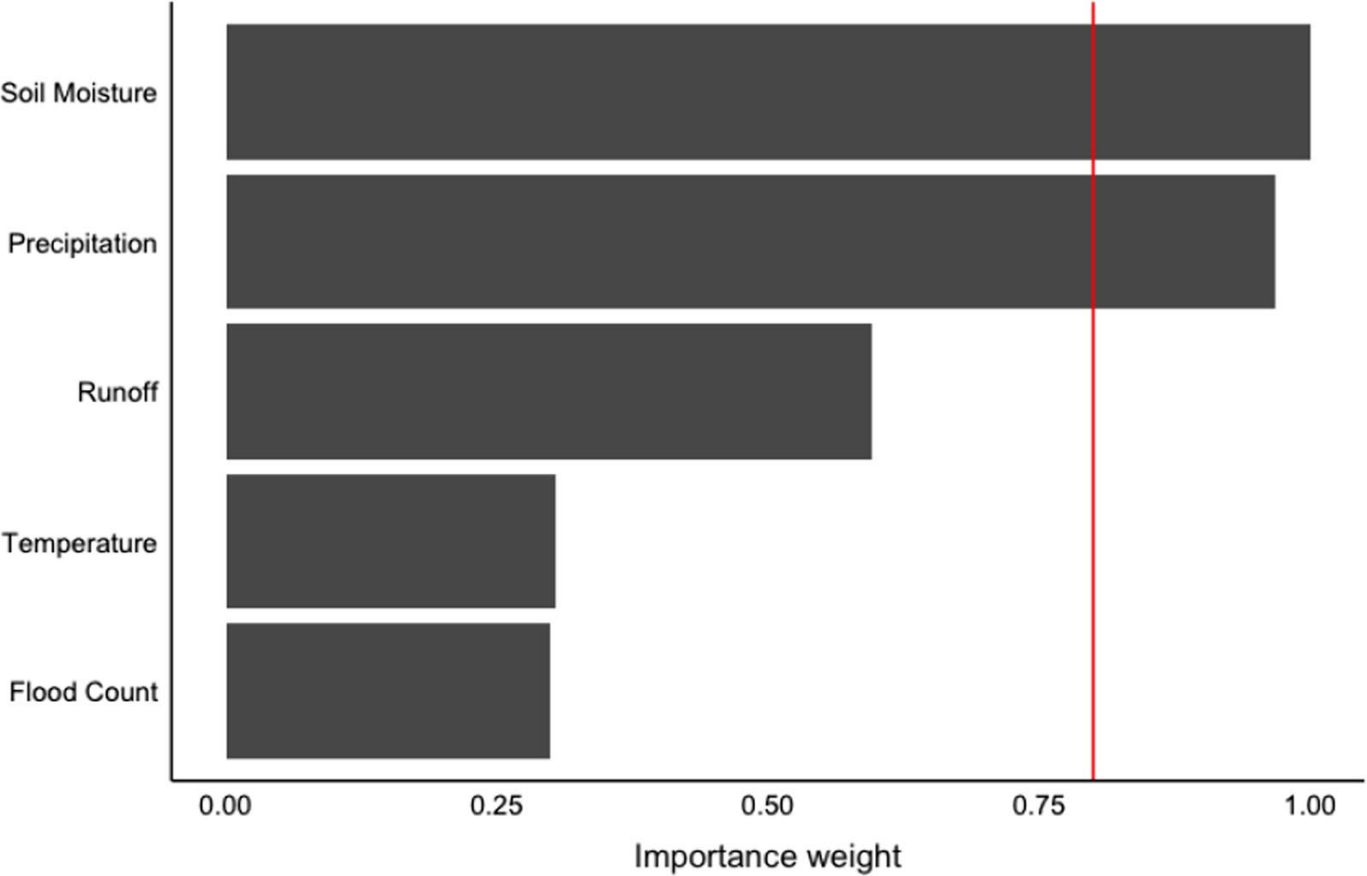
Soil moisture and precipitation were the most highly weighted flood-indicator variables assessed in the multimodel inference analysis; these variables were highly weighted in 98 and 96% of the candidate models, respectively. The red line indicates where variables are highly weighted in at least 80% of the candidate models; variables that exceed this importance threshold are included in the final model

Soil moisture and precipitation were the most highly weighted variables identified by the importance weighting and multimodel inference (Fig. 4). Both variables were positively associated with a significant increase in monthly Legionnaires’ disease hospitalizations and were included in all of the top models (Table 3). A 1-standard deviation increase in average soil moisture was associated with a 49% increase in hospitalizations in the most highly weighted model (Table 3). In the same model, a 1-standard deviation increase in average precipitation was associated with a 26% increase in hospitalizations. Temperature and the other hydrometeorological variables were not significantly associated with Legionnaires’ disease hospitalizations in any of the top models. The importance weights, top models, and effect estimates were consistent across all hospitalization thresholds (Additional file 1: Table S4) and in the cross-validation sensitivity analysis. Similarly, multimodel inference using flood-indicator data aggregated to the hospitals’ catchment areas identified the same top models and comparable effect estimates. In the most highly weighted model, a 1-standard deviation increase in soil moisture and precipitation at the catchment level was associated with a 53% and 26% increase in hospitalizations, respectively (Additional file 1: Table S5).

**Table 3:** Association between Legionnaires’ disease hospitalizations and meteorological variables in the most highly weighted models

Model	Precipitation	Soil moisture	Temperature	Runoff	Flood count	Model weight
1	0.26 (0.14, 0.38)	0.49 (0.24, 0.74)		− 0.08 (− 0.16, 0.00)		0.261
2	0.28 (0.16, 0.40)	0.48 (0.23, 0.73)		− 0.07 (− 0.13, − 0.01)	− 0.03 (− 0.11, 0.05)	0.176
3	0.19 (0.01, 0.29)	0.44 (0.20, 0.68)				0.133
4	0.23 (0.13, 0.33)	0.42 (0.17, 0.67)			− 0.04 (− 0.10, 0.02)	0.125
5	0.26 (0.14, 0.38)	0.47 (0.20, 0.74)	− 1.28 (− 6.49, 3.93)	− 0.08 (− 0.16, 0.00)		0.104
6	0.28 (0.16, 0.40)	0.50 (0.23, 0.77)	− 1.27 (− 6.54, 4.00)	− 0.07 (− 0.13, − 0.01)	− 0.04 (− 0.10, 0.02)	0.070
7	0.19 (0.01, 0.29)	0.42 (0.17, 0.67)	− 1.31 (− 6.74, 4.12)			0.053

Effect estimates are the change in monthly hospitalizations associated with a 1-standard deviation increase in the meteorological variables; values in parentheses indicate the 95% confidence interval

## Discussion

The incidence of sporadic Legionnaires’ disease has increased for over 20 years, but the association between cases and flooding as a potential driver of disease has not been thoroughly examined in the US. Previous studies have found positive associations among cases, rainfall, and relative humidity, but most have focused on specific cities or small geographic regions [10, 11, 60]. Many have also relied on weather data from a single source [28, 30] or a single source per state [29], which may obscure local variation in meteorological conditions. In this study, the association between flooding, measured by several hydrometeorological variables, and Legionnaires’ disease hospitalizations was analyzed across 75 hospitals in 25 states in the US, a geographic scope that encompasses a range of climatological regimes and demographics.

This work suggests that flooding, which can lead to the contamination of household and recreational water sources [37, 41], may be associated with hospitalizations for Legionnaires’ disease. While previous work has implied that rainfall influences the spread of disease via contamination [61, 62], none have focused on identifying or quantifying flood events as the driver of transmission. To address this gap, we used several methods to characterize extreme and seasonal floods; we found that hospitalizations increased during months with flooding due to extreme storms and were positively associated with monthly precipitation and soil moisture, which are common flood-indicator variables.

The seasonality and intensity of flooding varies considerably throughout the US, and these events cannot be measured with a single flood-indicator variable. We used two definitions of extreme events to account for the variety of flood types that occur, including those associated with hurricanes or tropical storms as well as those unrelated to cyclonic storms (e.g. due to intense precipitation and snowmelt). In the first approach, hospitalizations increased 32% in months with named storms during the Atlantic storm basin season among hospitals in the mid-Atlantic and Northeast. The second approach reinforced this finding and determined that hospitalizations throughout the US increased in months with anomalously high precipitation, not just those affected by cyclonic storms. The extreme event analysis supported a 2005 study that found legionellosis was positively associated with high atmospheric pressure more 10 days before occurrence and low atmospheric pressure within 5 days of occurrence, consistent with the transition that occurs when a storm front moves through an area [10].

In addition to extreme floods, many parts of the US experience seasonal flooding, including floods associated with snowmelt, frequent thunderstorms, and flash floods after droughts. We used multiple flood-indicator variables to characterize these seasonal floods and found that monthly soil moisture and precipitation are associated with increased Legionnaires’ disease hospitalizations. The association between rainfall and cases is well-supported, but this is the first to assess soil moisture, which functions as an integrator of rainfall and is an important flood indicator. *Legionella* bacteria thrive in extremely warm environments but in our analysis, extreme or seasonal temperature was not significantly associated with hospitalizations. This effect of temperature on Legionnaires’ disease is inconsistent with previous studies; temperature lagged from 1 to 9 weeks was predictive of cases in some studies [28, 31] and associated with a decrease in disease rates in others. Given laboratory studies demonstrating that *Legionella* bacteria preferentially grow at high temperatures, it is likely that environmental temperature influences transmission. Our findings suggest, however, that extreme or seasonal flood events are more strongly associated with increased hospitalizations whereas temperature alone is not.

While the effect of flooding on Legionnaires’ disease has not been examined in the US, our findings are supported by earlier research on the relationship between flood-indicator variables and outbreaks of other waterborne diseases [35, 46, 47]. An analysis of 42 years of outbreaks in the US found that 51% were preceded by extreme rainfall and that 60% were attributed to drinking water contamination [14]. This study did not examine the mechanisms by which flooding affects Legionnaires’ disease hospitalizations, but previous research has identified *Legionella* in environments that are vulnerable to flooding. *Legionella* have been detected in surface runoff [27, 61, 62], which can directly contaminate drinking water sources or overwhelm water treatment systems during floods. The bacteria have also been found in wastewater and sewage treatment plants [63, 64], which are prone to overflows and contamination events associated with floods [65]. Our findings indicate an association between flooding and Legionnaires’ disease, and future research should focus on examining the mechanisms by which flooding could lead to contamination and drive transmission.

Sources of contamination typical in Legionnaires’ disease outbreaks, namely cooling towers, plumbing systems, and recreational or decorative pools [66], are often not the source for sporadic cases [28]. Transmission of sporadic, community-acquired cases may instead be driven by household water and environmental exposures. Previous studies have attributed up to 40% of sporadic cases to potable water [67], and an elevated risk of infection has been associated with water from private wells [68] and from surface water (compared to potable water from groundwater sources) [62]. Water quality data from a range of sources could help determine the primary modes of exposure to *Legionella* for sporadic cases not associated with point source contamination. Detailed exposure analyses would also lead to an improved understanding of how infection occurs; while Legionnaires’ disease transmission is thought to occur primarily via the inhalation of aerosolized bacteria, some studies suggest that infection also occurs via aspiration [69, 70]. Contaminated drinking water may be a crucial source of exposure if infection occurs via aspiration, as aerosolization by a household item (e.g. a showerhead, faucet, or hose) would not be required for transmission.

Our findings are constrained by a number of limitations related to the availability and resolution of the hospitalization data. The analysis does not include any data from the Southeastern US because these states either do not contribute to the HCUP dataset or do not provide monthly data; this is a major limitation, as states in this region are most prone to cyclonic storms. However, regions with the highest incidence of Legionnaires’ disease were included in the analysis, and states that did not contribute to the HCUP dataset generally had lower incidence compared to the national average [71]. A recent analysis of Legionnaires’ disease epidemiological trends in the United States between 1992 and 2018 found that age-standardized average incidence was higher in the Northeast and Midwest compared to the South and West, and highest in New England and the Mid-Atlantic states [71]; these geographic differences in incidence were more pronounced later in the time series (after 2002), which overlaps with most of the study period in this analysis. Future studies should examine the associations among hydrometeorological conditions and Legionnaires’ disease throughout the US, particularly in the Southeast, but the regions included in the study capture the states with the highest Legionnaires’ disease burden.

Despite rising incidence, hospitalizations for Legionnaires’ disease are relatively uncommon and as such our study relies on a small number of cases. During the study period, the total number of annual cases, not just hospitalizations, in the US reported to the Centers for Disease Control and Prevention (CDC) ranged from 969 to 3,676 [72]. To address this limitation, we repeated the analysis using several case-count thresholds in order to examine the consistency of our findings when different hospitals were included in the dataset. The stability of the associations, even when hospitals with a single case were included in the dataset, indicate that the findings are robust.

The National Inpatient Sample only provides monthly hospitalization data, which prohibits a more temporally resolved analysis, and the geographic location of the hospital, not the residential locations of the cases. The absence of more temporally or geographically resolved data introduces the possibility of misclassification bias, given that the flood data associated with the hospital’s zip code may not accurately reflect the conditions at the cases’ residential zip codes. We aimed to address these limitations by including a large number of hospitals in the study from rural, urban, and suburban areas and evaluating the consistency of our findings across different study sites. Our findings are also consistent with small-scale studies that used daily case data [10] or had residential location data [33].

## Conclusion

Both seasonal and extreme flooding is projected to increase in conjunction with warming atmospheric temperatures, and our ability to mitigate the effect of these floods is contingent upon a thorough understanding of flood-disease dynamics and how they geographically vary. Our findings suggest that the increase of Legionnaires’ disease across the US may be explained by flooding and that mitigating the effects of these events in the future is key to reducing the spread of disease. These results also suggest that current flood or contamination control measures are insufficient with respect to *Legionella* and may indicate that more rigorous water and wastewater treatment policies are required. The findings may also be of use to clinicians treating patients with respiratory symptoms in the wake of extreme events or during seasonal flood periods. While awareness of and testing for legionnaires’ disease has increased, it remains substantially underdiagnosed and underreported among younger and immune-competent individuals. Future analysis should incorporate detailed water quality data from natural and built environments to better understand the routes of exposure, and how hydrological events affect transmission.

## Supplementary Information

**Additional file 1. **Legionnaires_Flooding_Supplement. **Table S1**. Description of HCUP hospitals grouped by Legionnaires’ disease case count thresholds. **Table S2**. Cyclonic storms that affected counties with HCUP hospitals between 2000 and 2011**. Table S3**. Percentile of average monthly hospitalizations in months with extreme meteorological conditions averaged across Hospital Service Areas (HSAs) compared to bootstrapped distribution of average monthly hospitalizations. **Table S4**. Association between Legionnaires’ disease hospitalizations and meteorological variables in the most highly weighted model for each hospitalization threshold. **Table S5**. Association between Legionnaires’ disease hospitalizations and meteorological variables averaged across Hospital Service Areas (HSAs) in the most highly weighted models**. Figure S1. a**–**b**. **a** Precipitation associated with cyclonic storms and **b** proximity to the storm track were not associated with a consistent significant change in monthly Legionnaires’ disease hospitalizations among hospitals that experienced the storms, compared to hospitals that were unexposed to the storms. Moderately intense precipitation (quartile 3) was associated with a significant increase in hospitalizations among the hospitals with a minimum of 15 and 20 total cases, but this association was insignificant at different precipitation levels and case thresholds. The analysis was restricted hospitals in regions that experience cyclonic storms from the Atlantic storm basin and to the months of the Atlantic storm season (June–November). **Figure S2.** **a**–**c** The association between exposure to cyclonic storms at the Hospital Service Area (HSA) level of analysis and monthly Legionnaires’ disease hospitalizations did not substantially differ from the associations identified using the county-level storm data. **a** Among hospitals in the 10-, 15-, and 20-case thresholds, hospitals in HSAs exposed to storms had a significant increase in hospitalizations compared to those in HSAs unexposed to storms. **b** Cyclonic-storm related precipitation and **c** proximity to storm tracks at the HSA level were not associated with significant changes in monthly hospitalizations; these findings are consistent with the analyses using county-level storm data (Fig. 2, Additional file 1: Fig. S1). **Figure S3. a**–**d** Monthly hydrometeorological flood-indicator variables averaged across the 75 hospitals in the primary analysis between 2000 and 2011, grouped by state (lines) and geographic region (color). The seasonality of **a** precipitation and **c** runoff differs in the Northeast and Midwest compared to the Southwest and West Coast, with peaks typically occurring in opposite months of the year. The seasonal pattern of **b** soil moisture and **d** streamflow is more consistent across the US, but the magnitude of the seasonal variation differs by region. **Model S1**. Description of negative binomial generalized mixed model used to assess the effect of cyclonic storms on hospitalizations. **Model S2**. Description of negative binomial generalized mixed model used to assess the effect of seasonal hydrometeorology and temperature on hospitalizations.

## Data Availability

The datasets supporting the conclusions of this article are available from HCUP (hospitalization data), NLDAS (meteorological data), NOAA (storm data), and USGS (stream flow data). HCUP data can be requested and purchased through the HCUP Central Distributor. The NLDAS, NOAA, and USGS datasets are publicly available and the sources are referenced in the paper.

## Abbreviations

AIC: Akaike information criterion
GLMM: Generalized linear mixed model
HCUP: Healthcare cost and utilization project
HSA: Hospital service area
NIS: National inpatient sample
NLDAS: NASA/NOAA North American land data assimilation system-2
NOAA: National oceanic and atmospheric administration
USGS: United States Geological Survey
