# Supplementary material for: The effect of seasonal and extreme floods on hospitalizations for Legionnaires’ disease in the United States, 2000–2011
Source: BMC Infect Dis. 2022 Jun 15;22:550. doi: 10.1186/s12879-022-07489-x (PMC9202215; doi:10.1186/s12879-022-07489-x)
Supplement: Supplementary file 1 — Additional file 1. Legionnaires_Flooding_Supplement. Table S1. Description of HCUP hospitals grouped by Legionnaires’ disease case count thresholds. Table S2. Cyclonic storms that affected counties with HCUP hospitals between 2000 and 2011. Table S3. Percentile of average monthly hospitalizations in months with extreme meteorological conditions averaged across Hospital Service Areas (HSAs) compared to bootstrapped distribution of average monthly hospitalizations. Table S4. Association between Legionnaires’ disease hospitalizations and meteorological variables in the most highly weighted model for each hospitalization threshold. Table S5. Association between Legionnaires’ disease hospitalizations and meteorological variables averaged across Hospital Service Areas (HSAs) in the most highly weighted models. Figure S1. a–b. a Precipitation associated with cyclonic storms and b proximity to the storm track were not associated with a consistent significant change in monthly Legionnaires’ disease hospitalizations among hospitals that experienced the storms, compared to hospitals that were unexposed to the storms. Moderately intense precipitation (quartile 3) was associated with a significant increase in hospitalizations among the hospitals with a minimum of 15 and 20 total cases, but this association was insignificant at different precipitation levels and case thresholds. The analysis was restricted hospitals in regions that experience cyclonic storms from the Atlantic storm basin and to the months of the Atlantic storm season (June–November). Figure S2. a–c The association between exposure to cyclonic storms at the Hospital Service Area (HSA) level of analysis and monthly Legionnaires’ disease hospitalizations did not substantially differ from the associations identified using the county-level storm data. a Among hospitals in the 10-, 15-, and 20-case thresholds, hospitals in HSAs exposed to storms had a significant increase in hospitalizations compared to those in HSAs u [file 12879_2022_7489_MOESM1_ESM.docx]

Supplementary Table 1. Description of HCUP hospitals grouped by Legionnaires’ disease case count thresholds

| LD Case Threshold | 1+ Case | 5+ Cases | 10+ Cases | 15+ Cases | 20+ Cases |
| --- | --- | --- | --- | --- | --- |
| Number of Hospitals | 378 | 151 | 75 | 36 | 25 |
| Number of LD Cases | 2,361 | 1,885 | 1,376 | 980 | 715 |
| Hospital Location (%) |  |  |  |  |  |
| Rural | 23.1 | 8.3 | 6.4 | 0 | 0 |
| Urban | 76.9 | 91.7 | 93.6 | 100 | 100 |
| Hospital Bedsize (%) |  |  |  |  |  |
| Small | 23 | 13.2 | 9.8 | 11.4 | 11.8 |
| Medium | 27.3 | 24.9 | 24.9 | 25.7 | 19.1 |
| Large | 49.6 | 61.9 | 65.4 | 62.9 | 69.1 |
| Geographic Region (%) |  |  |  |  |  |
| Northeast | 48 | 60.2 | 66.2 | 72.9 | 76.5 |
| Midwest | 25.4 | 18.2 | 16.8 | 2.9 | 16.2 |
| Southwest | 9 | 9.7 | 12.3 | 14.3 | 7.4 |
| West Coast | 17.6 | 11.9 | 4.7 | 0 | 0 |
| Mean Annual Discharge (SD) | 13,700 (11,200) | 20,400 (12,000) | 23,300 (13,100) | 26,100 (13,700) | 29,900 (14,000) |

Supplementary Table 2. Cyclonic storms that affected counties with HCUP hospitals between 2000 and 2011

| Storm | Year | Number of affected counties with HCUP hospitals |
| --- | --- | --- |
| Allison | 2001 | 46 |
| Charley | 2004 | 58 |
| Dennis | 2005 | 16 |
| Frances | 2004 | 32 |
| Gustav | 2008 | 31 |
| Ike | 2008 | 32 |
| Irene | 2011 | 41 |
| Isabel | 2003 | 22 |
| Isidore | 2002 | 20 |
| Ivan | 2004 | 37 |
| Jeanne | 2004 | 48 |
| Katrina | 2005 | 6 |
| Lili | 2002 | 2 |
| Noel | 2007 | 2 |
| Rita | 2005 | 4 |

Supplementary Table 3. Percentile of average monthly hospitalizations in months with extreme meteorological conditions averaged across Hospital Service Areas (HSAs) compared to bootstrapped distribution of average monthly hospitalizations

| Hospitalization Threshold | Precipitation | Runoff | Soil Moisture | Temperature |
| --- | --- | --- | --- | --- |
| 1+ Cases | 0.75 | 0.49 | 0.35 | 0.43 |
| 5+ Cases | 0.81 | 0.57 | 0.37 | 0.45 |
| 10+ Cases | 0.84 | 0.44 | 0.50 | 0.41 |
| 15+ Cases | 0.91 | 0.66 | 0.52 | 0.41 |
| 20+ Cases | 0.93 | 0.76 | 0.79 | 0.58 |

Supplementary Table 4. Association between Legionnaires’ disease hospitalizations and meteorological variables in the most highly weighted model for each hospitalization threshold

| Hospitalization Threshold | Precipitation | Soil moisture | Temperature | Runoff | Flood count | Model weight |
| --- | --- | --- | --- | --- | --- | --- |
| 1+ Case | 0.15 (0.09, 0.21) | 0.20 (0.10, 0.29) |  | -0.07 (-0.13, -0.01) |  | 0.21 |
| 5+ Cases | 0.19 (0.07, 0.31) | 0.55 (0.43, 0.67) |  | -0.06 (-0.14, 0.02) |  | 0.29 |
| 10+ Cases | 0.26 (0.14, 0.38) | 0.49 (0.24, 0.74) |  | -0.08 (-0.16, 0.00) |  | 0.26 |
| 15+ Cases | 0.37 (0.21, 0.53) | 0.61 (0.20, 1.02) |  | -0.10 (-0.20, 0.00) |  | 0.26 |
| 20+ Cases | 0.36 (0.18, 0.54) | 0.92 (0.35, 1.49) | -11.64 (-23.97, 0.69) | -0.10 (-0.22, 0.02) |  | 0.24 |

Note: Effect estimates are the change in monthly hospitalizations associated with a 1-standard deviation increase in the meteorological variables; values in parentheses indicate the 95% confidence interval.

Supplementary Table 5. Association between Legionnaires’ disease hospitalizations and meteorological variables averaged across Hospital Service Areas (HSAs) in the most highly weighted models

| Model | Precipitation | Soil moisture | Temperature | Runoff | Flood count | Model weight |
| --- | --- | --- | --- | --- | --- | --- |
| 1 | 0.26 (0.12, 0.40) | 0.53 (0.22, 0.85) |  | -0.08 (-0.16, -0.00) |  | 0.294 |
| 2 | 0.28 (0.14, 0.42) | 0.52 (0.21, 0.83) |  | -0.08 (-0.16, -0.00) | -0.03 (-0.09 0.03) | 0.179 |
| 3 | 0.19 (0.07, 0.31) | 0.48 (0.17, 0.79) |  |  |  | 0.125 |
| 4 | 0.22 (0.10, 0.34) | 0.48 (0.17, 0.79) |  |  | -0.04 (-0.10, 0.02) | 0.118 |
| 5 | 0.25 (0.11, 0.39) | 0.55 (0.22, 0.88) | 1.25 (-5.57, 8.07) | -0.08 (-0.16, -0.00) |  | 0.084 |
| 6 | 0.27 (0.13, 0.41) | 0.54 (0.21, 0.87) | 1.31 (-5.57, 8.19) | -0.08 (-0.16, -0.00) | -0.03 (-0.11, 0.05) | 0.079 |
| 7 | 0.18 (0.06, 0.30) | 0.50 (0.19, 0.81) | 1.38 (-5.62, 8.38) |  |  | 0.051 |

Note: Effect estimates are the change in monthly hospitalizations associated with a 1-standard deviation increase in the meteorological variables averaged across HSAs; values in parentheses indicate the 95% confidence interval.


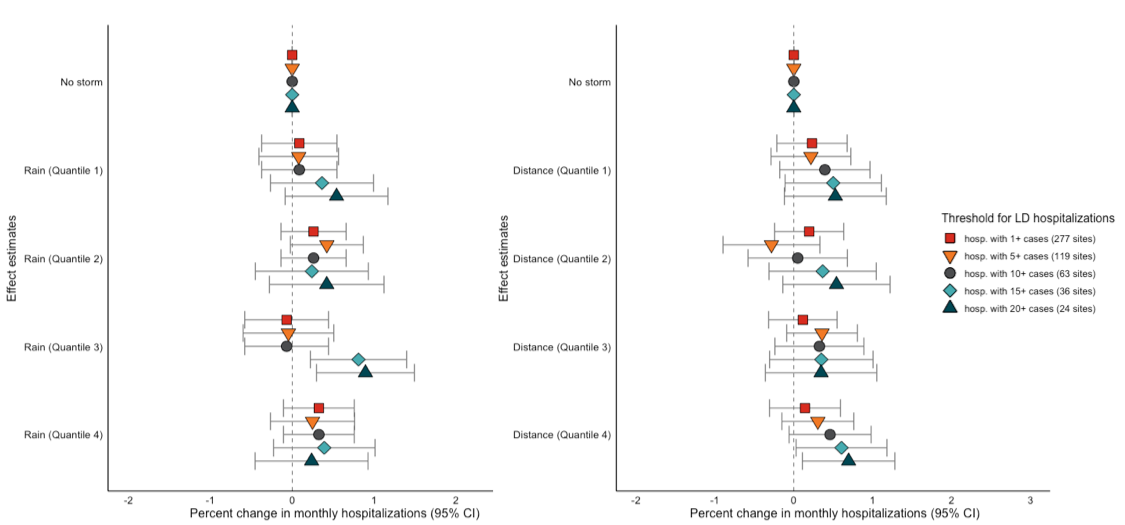


(b)

(a)

Supplementary Figure 1. a) Precipitation associated with cyclonic storms and b) proximity to the storm track were not associated with a consistent significant change in monthly Legionnaires’ disease hospitalizations among hospitals that experienced the storms, compared to hospitals that were unexposed to the storms. Moderately intense precipitation (quartile 3) was associated with a significant increase in hospitalizations among the hospitals with a minimum of 15 and 20 total cases, but this association was insignificant at different precipitation levels and case thresholds. The analysis was restricted hospitals in regions that experience cyclonic storms from the Atlantic storm basin and to the months of the Atlantic storm season (June – November).


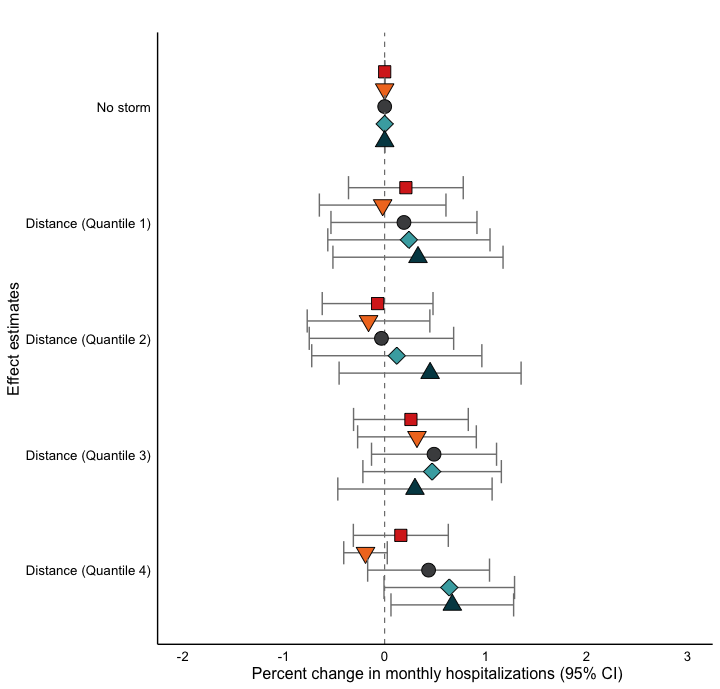

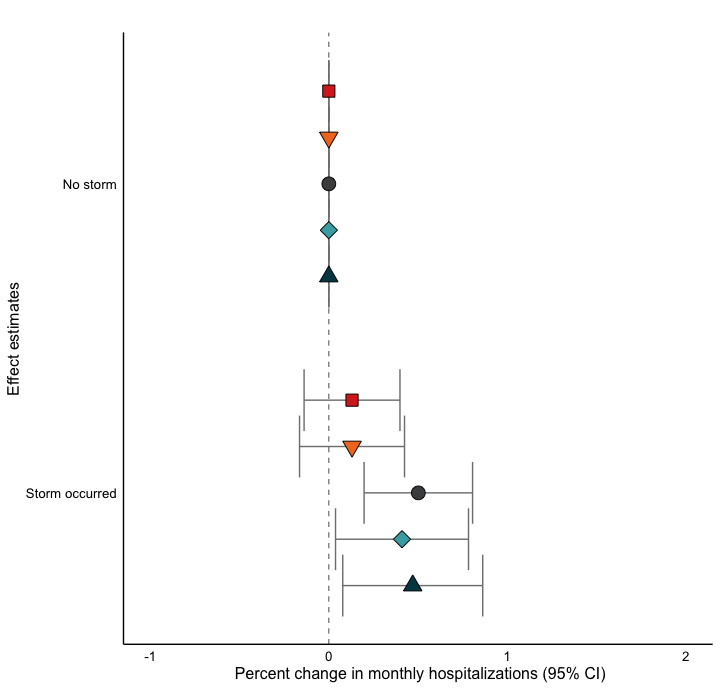

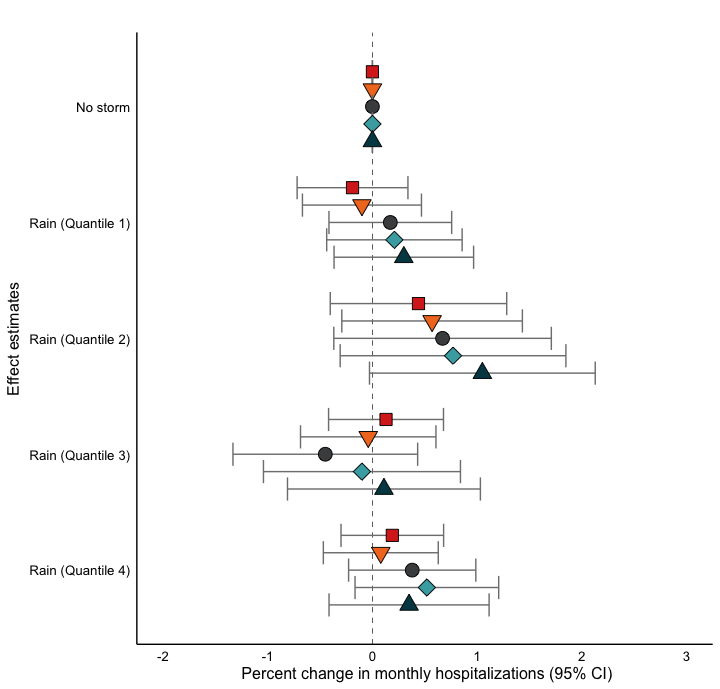

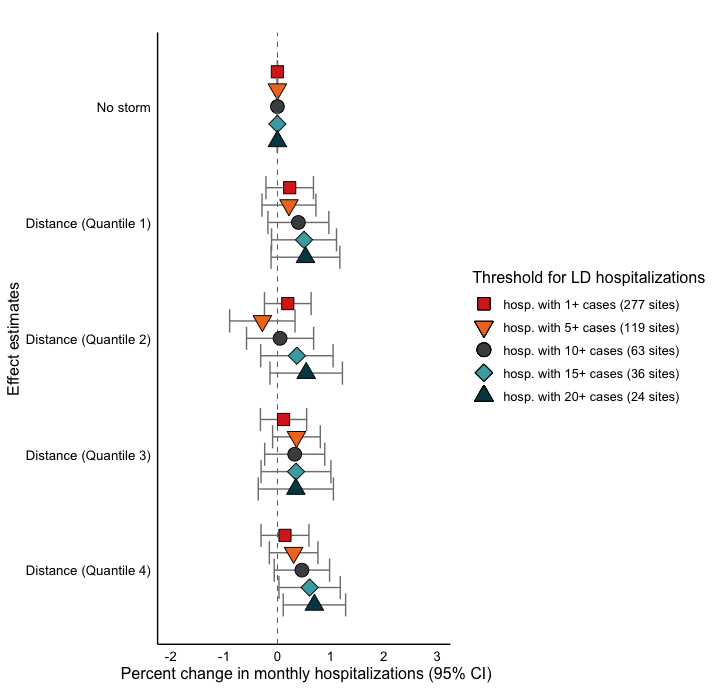


(c)

(a)

(b)

Supplementary Figure 2. The association between exposure to cyclonic storms at the Hospital Service Area (HSA) level of analysis and monthly Legionnaires’ disease hospitalizations did not substantially differ from the associations identified using the county-level storm data. a) Among hospitals in the 10-, 15-, and 20-case thresholds, hospitals in HSAs exposed to storms had a significant increase in hospitalizations compared to those in HSAs unexposed to storms. b) Cyclonic-storm related precipitation and c) proximity to storm tracks at the HSA level were not associated with significant changes in monthly hospitalizations; these findings are consistent with the analyses using county-level storm data (Fig.2, Supp. Fig. 1).


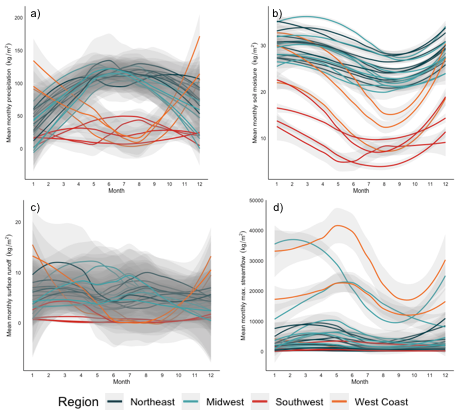


Supplementary Figure 3. Monthly hydrometeorological flood-indicator variables averaged across the 75 hospitals in the primary analysis between 2000 and 2011, grouped by state (lines) and geographic region (color). The seasonality of a) precipitation and c) runoff differs in the Northeast and Midwest compared to the Southwest and West Coast, with peaks typically occurring in opposite months of the year. The seasonal pattern of b) soil moisture and d) streamflow is more consistent across the US, but the magnitude of the seasonal variation differs by region.

Supplementary Model Description 1.

Observed number of hospitalizations in hospital *i*, Y*_i_*, is assumed to be distributed as a negative binomial variable:

Y*_i_* ~ NB (Y*_i_* | μ*_i_*, θ)

where μ*_i_* is the mean and θ is the shape parameter.

The model structure is:

log(μ*_i_*) = X*_i_*β + Z*_i_*b + log(P*_i_*)

Where X*_i_* are the variables of interest, which are storm occurrence and location, β is the vector of fixed effects for X*_i_*, b is the vector of random effects for sample variables Z*_i_*, and P*_i_* is the offset, which is the total monthly hospitalizations for hospital *i*. The vector of K random effects, b, is assumed to follow a normal distribution: b ~ N_k_ (0, Ψ) where Ψ is a positive definite variance-covariance matrix that determines the random effects. The statistical analysis was performed in R.

Supplementary Model Description 2.

Observed number of hospitalizations in hospital *i* at month *t*, Y*_it_*, is assumed to be distributed as a negative binomial variable:

Y*_it_* ~ NB (Y*_it_* | μ*_it_*, θ)

where μ*_it_* is the mean, θ is the shape parameter and *t* = 1,…, *n*. Here *n* (144) is the number of months in the study period.

The model structure is:

log(μ*_it_*) = X*_it_*β + Z*_it_*b + log(P*_it_*)

Where X*_it_* are the variables of interest, which are the monthly standardized average hydrometeorological and temperature variables, monthly sine and cosine terms to account for seasonality (sin(2π x $\frac{\mathrm{month}_{t}}{12}$) and cos(2π x $\frac{\mathrm{month}_{t}}{12}$)), and a term for year to account for long-term trends. β is the vector of fixed effects for X*_it_*, b is the vector of random effects for sample variables Z*_it_*, and P*_it_* is the offset, which is the total monthly hospitalizations for hospital *i* in time *t*. The vector of K random effects, b, is assumed to follow a normal distribution: b ~ N_k_ (0, Ψ) where Ψ is a positive definite variance-covariance matrix that determines the random effects. The statistical analysis was performed in R.
